# *In Situ* Characterization of Mixtures of Linear and Branched Hydrocarbons Confined within Porous Media Using 2D DQF-COSY NMR Spectroscopy

**DOI:** 10.1021/acs.analchem.1c04295

**Published:** 2022-02-12

**Authors:** Qingyuan Zheng, Mick D. Mantle, Andrew J. Sederman, Timothy A. Baart, Constant M. Guédon, Lynn F. Gladden

**Affiliations:** † Department of Chemical Engineering and Biotechnology, University of Cambridge, West Cambridge Site, Philippa Fawcett Drive, Cambridge CB3 0AS, U.K.; ‡ Shell Global Solutions International B.V., Grasweg 31, Amsterdam 1031 HW, The Netherlands

## Abstract

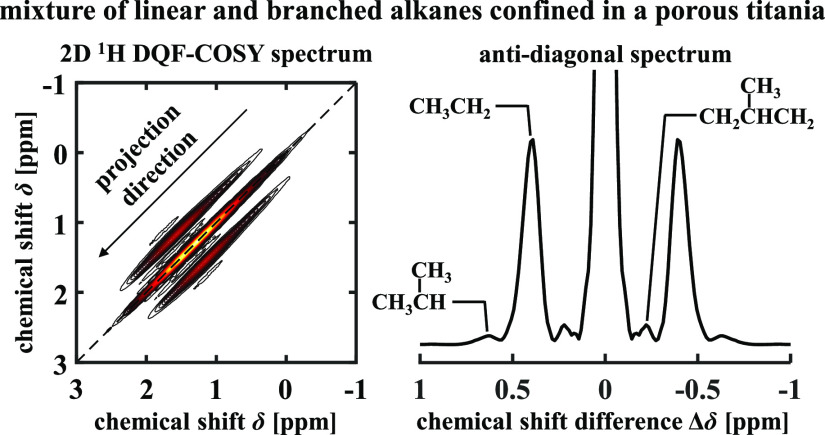

The analysis of 1D anti-diagonal spectra from the projections of 2D double-quantum filtered correlation spectroscopy NMR spectra is presented for the determination of the compositions of liquid mixtures of linear and branched alkanes confined within porous media. These projected spectra do not include the effects of line broadening and therefore retain high-resolution information even in the presence of inhomogeneous magnetic fields as are commonly found in porous media. A partial least-square regression analysis is used to characterize the mixture compositions. Two case studies are considered. First, mixtures of 2-methyl alkanes and *n*-alkanes are investigated. It is shown that estimation of the mol % of branched species present was achieved with a root-mean-square error of prediction (RMSEP) of 1.4 mol %. Second, the quantification of multicomponent mixtures consisting of linear alkanes and 2-, 3-, and 4-monomethyl alkanes was considered. Discrimination of 2-methyl and linear alkanes from other branched isomers in the mixture was achieved, although discrimination between 3- and 4- monomethyl alkanes was not possible. Compositions of the linear alkane, 2-methyl alkane, and the total composition of 3- and 4-methyl alkanes were estimated with a RMSEP <3 mol %. The approach was then used to estimate the composition of the mixtures in terms of submolecular groups of CH_3_CH_2_, (CH_3_)_2_CH, and CH_2_CH(CH_3_)CH_2_ present in the mixtures; a RMSEP <1 mol % was achieved for all groups. The ability to characterize the mixture compositions in terms of molecular subgroups allows the application of the method to characterize mixtures containing multimethyl alkanes. The motivation for this work is to develop a method for determining the mixture composition inside the catalyst pores during Fischer–Tropsch synthesis. However, the method reported is generic and can be applied to any system in which there is a need to characterize mixture compositions of linear and branched alkanes.

## Introduction

The ability to characterize the composition of a reaction mixture inside the pore space of a catalyst provides a powerful tool to probe how the catalyst formulation and pore structure and reactor operating conditions influence the product composition and hence provides insight into catalyst performance. This work reports an experimental methodology for characterizing the hydrocarbon mixtures forming inside catalyst pores with a particular focus on discriminating linear and branched hydrocarbons. This is of particular interest with regard to gaining insight into the catalytic mechanism and the resulting product composition.

Analytical techniques such as gas chromatography,^1,2^ mass spectroscopy,^3,4^ and nuclear magnetic resonance (NMR)^5−10^ spectroscopy are routinely used to characterize hydrocarbon mixtures in the bulk liquid phase; in the context of catalysts and catalytic processes, such mixtures might be the liquid product of the catalytic reaction or the liquid mixture extracted from the pore space. With regard to NMR, 1D ^13^C NMR^5−8^ and ^1^H 2D correlation spectroscopy (COSY)^10^ have been applied to characterize the types and compositions of branched alkanes in bulk liquid mixtures of hydrocarbons. More generally, 2D NMR spectroscopy is established as a powerful tool in identifying the molecular structure and thus discriminating chemical species in complex mixtures.^11−13^ However, the application of NMR to characterize such mixtures inside the pores of heterogeneous catalysts is significantly hindered by line broadening of the NMR signal due to the enhanced field inhomogeneity characteristic of porous media. Application to the characterization of liquid mixtures and, in particular, the identification of branched hydrocarbon species while inside catalyst pores has been reported. One such example is the measurement of *iso*-butane composition during *n*-butane isomerization.^9^ However, to achieve this measurement, ^13^C magic-angle spinning solid-state NMR was employed, which cannot be used in studying catalysts working inside a packed-bed reactor operating under industrially relevant conditions. Recently, Terenzi *et al.*(14) applied COSY to determine the carbon number of pure *n*-alkanes and *n*-alkane mixtures confined within a porous titania. The 2D COSY spectra were projected along the diagonal direction to form anti-diagonal projected spectra. It was reported that while line broadening was observed along the main diagonal direction of the 2D spectra, the line-broadening effect was not observed in the anti-diagonal direction and therefore in the anti-diagonal projected spectra.^14^ This absence of line broadening is explained by the fact that spectral properties perpendicular to the diagonal are determined by intramolecular properties, such as the chemical shift difference Δδ between coupled ^1^H nuclei and the corresponding *J*-coupling constants, which remain unchanged in the presence of magnetic field inhomogeneity. Therefore, high spectral resolution is retained in the 1D projection along the main diagonal direction; herein, we refer to the 1D projection of the main diagonal as the anti-diagonal spectrum. As a result, the 1D anti-diagonal spectrum of a 2D COSY experiment can be used for the quantitative analysis of mixtures confined within porous media.

While the 2D COSY pulse sequence should, in principle, enable acquisition of the information required for the characterization of the liquid mixtures inside the pore space, the standard COSY pulse sequence is limited in its application because of the intense diagonal signal which suffers from significant *t*_1_ noise.^11,15^ As a result, the analysis of cross-peaks, which contain the information on the molecular structure needed to discriminate branched and linear alkanes, can be significantly obscured. To overcome this problem, the ^1^H double-quantum filtered (DQF) COSY technique^11,16^ is used. The DQF-COSY pulse sequence yields an anti-phase multiplet signal along the main diagonal;^11^ the positive and negative parts of these anti-phase multiplets act to cancel out each other and result in the suppression of the signal along the main diagonal and the associated *t*_1_ noise. Apart from reduced signal intensities due to the application of the double-quantum filter,^11^ the characteristics of DQF-COSY cross-peaks, and hence the associated analysis of the spectra, are the same as those acquired with COSY.

In this work, 2D ^1^H DQF-COSY spectroscopy is used to enable the discrimination and quantification of the chemical species in mixtures confined in porous media. Such measurement would be impossible to achieve by 1D NMR spectroscopy because of the line-broadening effect. The method is demonstrated in application to the characterization of mixtures of branched and linear alkanes. The measurement is of immediate relevance to Fischer–Tropsch (FT) synthesis, although the characterization of branched alkanes within *n*-alkane mixtures is also of relevance to catalytic hydrocracking and isomerization of alkanes.^17,18^ FT synthesis converts hydrogen and carbon monoxide to hydrocarbon mixtures which are subsequently used to produce synthetic fuels and lubrication oils.^19^ In conventional FT synthesis, while the majority of products are linear *n*-alkanes, branched alkanes are produced in small amounts, typically <20%, in product mixtures. Monomethyl branched alkanes have been reported as the predominant branching products, and different branched isomers can coexist with the branching methyl group distributed along the alkyl chains.^1,7,20,21^ Recent studies using bifunctional catalysts^22,23^ have reported product compositions containing as much as >60 mol % branched alkanes. Being able to control the amount and nature of branched alkanes enables control over the properties and quality of fuels.^22,24,25^ Furthermore, the branching compositions in product mixtures have been reported to reflect the reaction mechanism of FT synthesis.^20,21^

The present study reports the first *in situ* quantification of branched alkanes from within the pores of a catalyst support. The extent to which DQF-COSY can characterize the nature and extent of branching of alkanes within the pores of a real catalyst support is studied. A partial least-square regression (PLSR) analysis was applied to estimate the mixture compositions from the DQF-COSY spectral data. First, a simple case of mixtures of 2-methyl alkanes and *n*-alkanes is considered, in which the molecular components are identified unambiguously from their spectral signatures. Second, the approach is applied to mixtures comprising linear alkanes and monomethyl alkanes, in particular, 2-methyl alkanes, 3-methyl alkanes, and 4-methyl alkanes. In this second case, the PLSR approach was further extended to characterize the mixtures in terms of the composition of the submolecular groups [CH_3_CH_2_, (CH_3_)_2_CH, and CH_2_CH(CH_3_)CH_2_] present in the mixtures.

## Experimental Section

### Materials

The liquid alkanes 2-methylheptane (2-C_7_, purity ≥99%), 3-methylheptane (3-C_7_, purity ≥97%), 2-methylnonane (2-C_9_, purity ≥98%), *n*-decane (*n*-C_10_, purity ≥99%), and *n*-dodecane (*n*-C_12_, purity ≥99%) were purchased from Fisher Scientific. 4-Methylnonane (4-C_9_, purity ≥98%) and *n*-hexadecane (*n*-C_16_, purity ≥99%) were purchased from Sigma-Aldrich. Measurements were made on the liquids confined within porous titania (TiO_2_) pellets, provided by Evonik Industries AG, in the form of cylindrical extrudates of a length and a diameter of 5 and 1.8 mm, respectively. The surface area, pore diameter, and pore volume of the titania were measured by nitrogen sorption analysis as 54.4 m^2^ g^–1^, 25.0 nm, and 0.34 cm^3^ g^–1^, respectively. The titania had a porosity of 0.58, as measured by mercury porosimetry.

### Experiments on the Mixtures of Branched and Linear Alkanes

The PLSR models were calibrated to estimate the mixture compositions using linear combinations of the spectral data of single-component 2-C_7_, 3-C_7_, 4-C_9_, and *n*-C_12_ liquid samples. The samples were prepared by filling a 5 mm NMR tube to a height of 10 mm; this height ensured that the entire sample sat within the length of the signal detection region of the radio frequency (r.f.) coil; hence, the liquid volume of the sample can be estimated from the acquired NMR signal (Supporting Information, section S1).

Test mixtures (TM1–19) were prepared with the compositions as reported in Tables 1 and S1. For samples TM12–TM19, the relative compositions between branched alkanes were consistent with those reported for the FT reaction on cobalt catalysts.^21^ The preparation of the samples of bulk liquid mixtures was the same as for pure bulk liquids. To prepare samples of liquid mixtures confined in the titania, the liquid mixture was imbibed in 3.6–4 g of the as-received titania pellets. To minimize the change of mixture composition due to competitive adsorption, a liquid mixture of the same volume as the total pore volume of the titania pellets was used to saturate the pellets so that all the liquid was imbibed within the pores. Therefore, the compositions of confined mixtures were considered the same as those of the prepared bulk liquid mixtures. The amounts of hydrocarbons required to prepare each liquid mixture were determined gravimetrically using a Precisa 125 A balance, measured to an accuracy of ±0.0001 g. The saturated titania pellets were packed into a glass tube of an inner diameter of 11 mm to a height of 38 mm.

**Table 1:** Estimation of the Compositions of Chemicals and Submolecular Groups in the Samples TM1–TM19 Using the PLSR Models^a^

	chemical composition [mol %]			group composition [mol %]		
samples	linear	2-methyl	3- + 4-methyl	group 1	group 2	group 3
TM1	95.2 (94.4)	4.8 (5.6)	0	97.6 (97.2)	2.4 (2.8)	0
TM2	92.0 (90.2)	8.0 (9.8)	0	96.0 (95.1)	4.0 (4.9)	0
TM3	86.2 (84.6)	13.8 (15.4)	0	93.1 (92.3)	6.9 (7.7)	0
TM4	81.7 (80.0)	18.3 (20.0)	0	90.8 (90.0)	9.2 (10.0)	0
TM5	58.5 (60.0)	41.5 (40.0)	0	79.2 (80.0)	20.8 (20.0)	0
TM6	38.1 (40.0)	61.9 (60.0)	0	69.1 (70.0)	31.0 (30.0)	0
TM7	–0.5 (0)	100.5 (100.0)	0	49.8 (50.0)	50.3 (50.0)	0
TM8	89.7 (89.2)	10.3 (10.8)	0	94.8 (94.6)	5.2 (5.4)	0
TM9	88.6 (89.6)	11.4 (10.4)	0	94.3 (94.8)	5.7 (5.2)	0
TM10	30.9 (32.9)	30.3 (33.4)	38.8 (33.7)	67.1 (66.4)	14.4 (16.7)	18.5 (16.9)
TM11	29.1 (24.5)	24.6 (25.2)	46.3 (50.2)	67.7 (66.5)	11.0 (11.2)	21.3 (22.3)
TM12	60.7 (60.0)	7.4 (8.6)	32.0 (31.5)	81.2 (81.1)	3.7 (4.0)	15.1 (14.8)
TM13	70.3 (70.1)	8.9 (9.4)	20.8 (20.4)	86.5 (85.7)	4.0 (4.5)	9.5 (9.8)
TM14	79.2 (78.0)	3.5 (3.8)	17.3 (18.2)	90.6 (89.4)	1.6 (1.8)	7.8 (8.8)
TM15	82.7 (87.8)	4.8 (3.7)	12.4 (8.4)	92.1 (94.0)	2.2 (1.8)	5.7 (4.2)
TM16	63.4 (59.9)	8.9 (7.9)	27.7 (32.2)	81.7 (81.1)	4.5 (3.7)	13.9 (15.2)
TM17	70.0 (69.6)	10.0 (8.9)	20.0 (21.5)	85.1 (85.4)	4.9 (4.3)	9.9 (10.3)
TM18	79.8 (79.4)	4.7 (4.1)	15.6 (16.4)	89.9 (90.0)	2.4 (2.0)	7.8 (8.0)
TM19	86.6 (90.0)	3.6 (2.8)	9.8 (7.2)	93.3 (95.1)	1.8 (1.4)	4.9 (3.6)

a The CH_3_CH_2_, (CH_3_)_2_CH, and CH_2_CH(CH_3_)CH_2_ groups are denoted as groups 1, 2, and 3, respectively, in the table. The estimated chemical and group compositions have standard errors of ±0.4 and ±0.2 mol %, respectively. The values in brackets are the compositions measured gravimetrically. Samples TM10–TM15 are prepared as bulk liquid mixtures. Samples TM1–TM9 and TM16–TM19 are prepared as liquid mixtures confined within the porous titania.

### NMR Experiments

The NMR measurements were performed on a Bruker AV 300 spectrometer which had a vertical superwide-bore 7.1 T superconducting magnet and a three-axis gradient set with a maximum gradient strength of 81 G cm^–1^ in each direction. The signal was detected using a r.f. coil of 66 mm inner diameter, which was tuned to the ^1^H resonance frequencies of the chemicals used. The typical 90° pulse length was 95 μs.

The 2D ^1^H DQF-COSY measurements were performed using a gradient-selective pulse sequence (cosygpmfqf in Topspin, Bruker). In this sequence, the selective acquisition of signals associated with double-quantum coherence was achieved by applying three sine-shaped gradient pulses of a duration of 1 ms and a gradient stabilization time of 200 μs along the vertical *z* direction. The relative gradient strength of the three gradient pulses in the order in which they were applied was 16:12:40. The acquisition was carried out with 8–16 scans with a recycle time of 10 s. Given that the *T*_1_ values of all the species studied were ≤2.5 s, the effect of *T*_1_ relaxation on the measurement is negligible. The time domain dataset consisted of 1024 × 256 complex points in the direct and indirect dimensions, and a sweep width of 4000 Hz was used in both dimensions. The dataset for each sample was acquired 2–3 times to estimate the experimental error.

### Data Analysis

#### Processing of DQF-COSY Data

**Figure 1:**
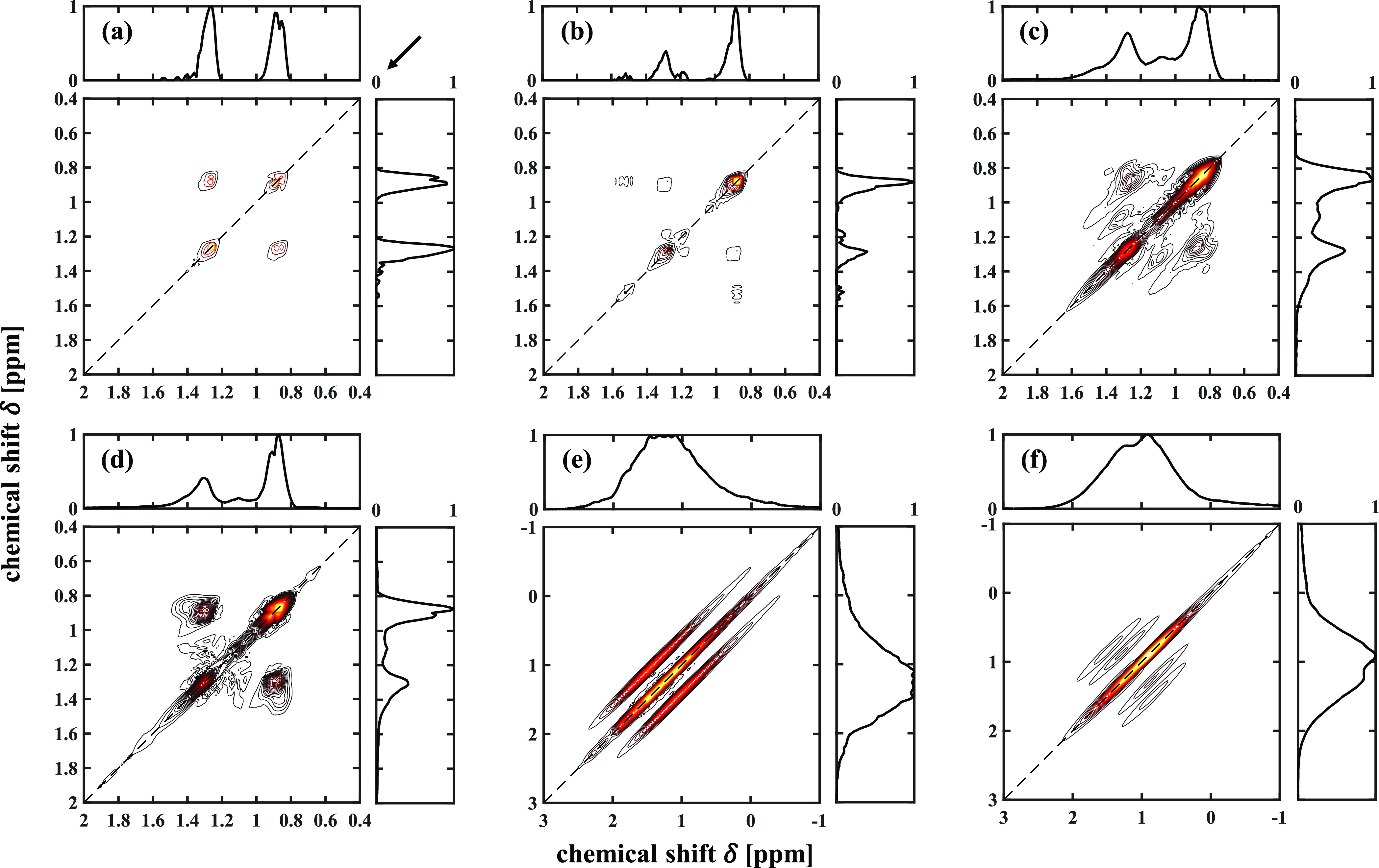
2D ^1^H DQF-COSY spectra of bulk liquids of (a) *n*-C_12_, (b) 2-C_7_, (c) 3-C_7_, (d) 4-C_9_, and of (e) *n*-C_12_ and (f) 2-C_7_ confined in the titania. The contour level for each 2D spectrum is different to allow clear visualization. The arrow at the top-right corner of (a) indicates the direction of the main diagonal projection that is used to obtain the 1D anti-diagonal spectra.

The time-domain dataset was first multiplied by the sine window functions in both dimensions, and the indirect dimension was then zero-filled to 1024 points. The 2D dataset was Fourier-transformed to obtain the 2D spectrum which was then modulus-corrected. The modulus-corrected spectrum was symmetrized against the diagonal of the spectrum, and baseline correction was then applied. The quantitative analysis of the data was performed on the 1D anti-diagonal spectra. The direction of the projection is indicated by the arrow at the top-right corner of Figure 1a.

#### Analysis of DQF-COSY Spectra

In a 2D DQF-COSY spectrum, the peaks located on the diagonal (dashed lines in Figure 1) are conventionally referred to as diagonal peaks and the peaks off the diagonal are known as cross-peaks. Cross-peaks are associated with pairs of ^1^H nuclei interacting by *J*-coupling and therefore contain the structural information characterizing a molecule. The intensity of a given cross-peak is associated with the number of nuclei involved in the coupling and the strength of *J*-coupling. The analysis focused on the cross-peaks. Only the cross-peaks associated with *J*-coupling between ^1^H nuclei within a distance of three chemical bonds were considered, as the *J*-coupling constants and the corresponding cross-peaks for coupling more than three bonds are negligible.^26^

Figure 1a–d shows the 2D DQF-COSY spectra of the bulk liquids of *n*-C_12_, 2-C_7_, 3-C_7_, and 4-C_9_, respectively, along with the standard 1D spectral projections of the 2D data onto the vertical and horizontal chemical shift axes. All the chemical shifts in this work are relative to the ^1^H resonance frequency of tetramethylsilane. The chemical shifts of ^1^H nuclei involved in *J*-coupling and the coordinates of the resulting cross-peaks in the 2D spectra in Figure 1 are summarized in Table S2. Figure 1a–d and Table S2 confirm that different types of branching lead to different cross-peaks, which discriminate linear and branched alkanes and different branched isomers. Figure 1e,f presents the DQF-COSY spectra, along with the standard 1D spectral projections on the vertical and horizontal axes, of *n*-C_12_ and 2-C_7_, respectively, confined within the porous titania. The cross-peaks observed in Figure 1a,b for bulk liquid species are again observed in Figure 1e,f, but the broadening along the main diagonal due to magnetic field inhomogeneity is also clearly seen. Figure 1e,f also confirms that it is impossible to discriminate the two species from their 1D spectra, as seen from the projected spectra.

**Figure 2:**
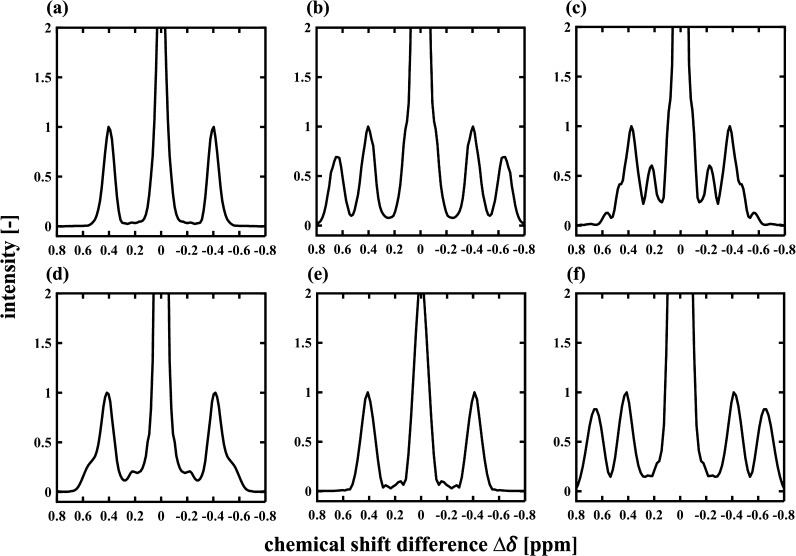
1D anti-diagonal spectra obtained from the data shown in Figure 1. The anti-diagonal spectra for bulk liquids of *n*-C_12_, 2-C_7_, 3-C_7_, and 4-C_9_ and confined liquid of *n*-C_12_ and 2-C_7_ are shown in (a–f), respectively. Each spectrum was normalized to the intensity of the peak located at Δδ ∼ 0.40 ppm.

Figure 2 presents the 1D anti-diagonal spectra of the 2D data shown in Figure 1. The horizontal axis of the 1D anti-diagonal spectra shows the chemical shift difference Δδ = δ_*x*_ – δ_*y*_, where δ_*x*_ and δ_*y*_ denote the coordinates of the 2D spectrum. For the signal located at the position [δ_*x*_, δ_*y*_] on the 2D spectrum, the anti-diagonal spectrum of the signal appears at Δδ = δ_*x*_ – δ_*y*_, which is proportional to the distance of the signal away from the main diagonal of the 2D data (|δ_*x*_ – δ_*y*_|/√2). Therefore, for the 1D anti-diagonal spectra, the peaks located at Δδ = 0 ppm correspond to the 2D main diagonal peaks while those located at Δδ ≠ 0 ppm correspond to 2D cross-peaks. The values of Δδ for cross-peaks are listed in Table S2. In Figure 2, the cross-peaks observed at Δδ ∼ ±0.40 ppm for all the species correspond to the CH_3_CH_2_ group at the linear terminus of an alkyl chain. The cross-peaks that allow discrimination of 2-C_7_ are located at Δδ = ±0.65 ppm (Figure 2b), which are associated with the (CH_3_)_2_CH group. 3-C_7_ and 4-C_9_ can be discriminated from *n*-C_12_ and 2-C_7_ by the cross-peaks at Δδ = ±0.22 ppm and Δδ = ±0.47–0.56 ppm (Figure 2c,d), which are associated with the CH_2_CH(CH_3_)CH_2_ groups. Discrimination between 3-C_7_ and 4-C_9_ is achieved in the bulk liquid state (Figure 1), for example, by the cross-peak associated with carbon [1, 2′] for 3-C_7_ (Table S2). However, this discrimination is impossible when these species are confined within the pore space of the titania because of line broadening along the main diagonal. The anti-diagonal spectra for *n*-C_12_ and 2-C_7_ confined in the titania are shown in Figure 2e,f, respectively. It is observed in these cases that the high chemical resolution is retained in the anti-diagonal spectra, consistent with the data acquired for those liquids in the bulk liquid state shown in Figure 2a,b. These anti-diagonal spectra are used to quantify the composition of the liquid mixtures.

#### PLSR Analysis

PLSR is well established as a multivariate calibration method that has been widely applied to the analysis of spectroscopy data.^27−29^ In this work, PLSR was applied to obtain calibration relationships between the DQF-COSY spectral data and mixture compositions. Given the complexity of the liquid mixtures that this approach will be used for when studying real catalytic data, the spectra upon which the PLSR models were calibrated were produced by linear combination of the spectra of the pure bulk-liquid species from which the mixtures were derived. For the initial analysis of mixtures of *n*-C_12_ and 2-C_7_, 11 calibration spectra were simulated based on the spectra of the two pure single-component species. For the four-component mixtures of *n*-C_12_, 2-C_7_, 3-C_7_, and 4-C_9_, the spectral data of the pure species were employed to simulate 1111 spectra for PLSR calibration. The details of the simulation of spectra for the calibration mixtures and the details of the PLSR calibration are given in the Supporting Information (sections S1 and S2, respectively).

The performance of the calibration models, and hence the error quoted for the resulting compositions, was evaluated using root-mean-square error (RMSE) and absolute error. The RMSEs calculated for the calibration and test samples are referred to as RMSEC and RMSEP, respectively. The details of the error analysis are reported in the Supporting Information (section S3).

## Results and Discussion

### Composition Analysis of the Mixtures of Linear and 2-Methyl Alkanes (TM1–TM9)

The results of the PLSR analysis are reported in Table 1. The RMSEPs of 2-methyl and linear alkanes were both calculated as 1.4 mol %, indicating accurate estimation. The RMSEC of the PLSR model was calculated as 0.8 mol %. It is noted that this approach is designed to characterize the extent and nature of branching in mixtures of alkanes and does not characterize the alkane chain length. In earlier work, the use of COSY to characterize the chain length of *n*-alkanes has been addressed, the analysis being based on the relative intensity between cross- and diagonal peaks of COSY spectra.^14^

**Figure 3:**
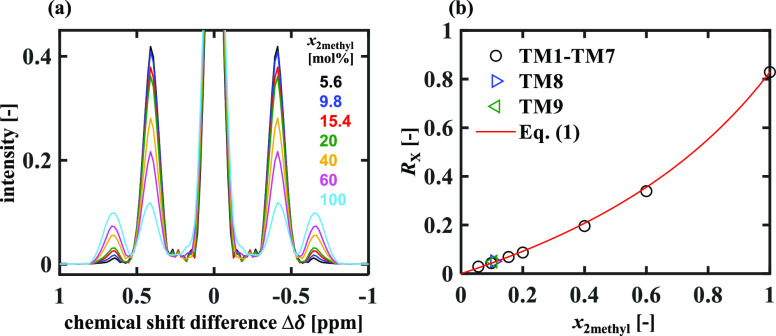
(a) Anti-diagonal spectra of mixtures TM1–TM7 with the 2-methyl alkane composition *x*_2methyl_ = 5.6–100 mol %. Each spectrum was normalized to the intensity at Δδ = 0 ppm. (b) Ratios of cross-peak intensities *R*_X_ against *x*_2methyl_. The *R*_X_ values have a standard error of ±0.001.

For the relatively simple application to the characterization of the amount of 2-methyl alkane species present in a mixture with *n*-alkanes, it is also possible to identify a simple theoretical relationship from which the 2-methyl alkane composition can be estimated from a single measurement of the ratio of the intensities of the cross-peaks characterizing the CH_3_CH_2_ and (CH_3_)_2_CH groups present in the mixture. Figure 3a shows the anti-diagonal spectra obtained from the binary mixtures of 2-C_7_ and *n*-C_12_ (TM1–TM7) in which the 2-C_7_ composition, *x*_2methyl_, lies in the range 5.6–100 mol %. Each spectrum in Figure 3a was normalized to its own maximum at Δδ = 0 ppm. It is observed that the cross-peaks at Δδ = ±0.40 ppm and Δδ = ±0.65 ppm associated with the CH_3_CH_2_ and (CH_3_)_2_CH groups, respectively, are well separated with their intensities identified unambiguously. Denoting the cross-peaks at Δδ = ±0.40 ppm and Δδ = ±0.65 ppm as cross-peak 1 (X1) and cross-peak 2 (X2), respectively, it is observed in Figure 3a that as *x*_2methyl_ increases, the intensities of X1 decrease, while the intensities of X2 increase. Given that one *n*-C_12_ molecule has two CH_3_CH_2_ groups and that one 2-C_7_ molecule has one CH_3_CH_2_ group and one (CH_3_)_2_CH group, a theoretical relationship between *x*_2methyl_ and the relative cross-peak intensity *R*_X_ = *I*_X2_/*I*_X1_ is obtained
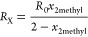
1where *R*_0_ = *I*_(CH_3_)_2_CH_/*I*_CH_3_CH_2__ is the intensity ratio of equal moles of the two groups. The *R*_0_ value was estimated from the pure-component spectrum (*x*_2methyl_ = 1), consistent with the PLSR approach, resulting in *R*_0_ = 0.829 ± 0.001. Figure 3b shows the *R*_X_ values calculated for samples TM1–TM9 plotted against *x*_2methyl_. Equation 1 is plotted as the solid line in Figure 3b, and it is observed that eq 1 estimates *x*_2methyl_ accurately with the RMSEP calculated as 1.1 mol %, similar to that for PLSR analysis.

### Characterization of the Mixtures of Linear and 2-, 3-, 4-Monomethyl Alkanes (TM10–TM19)

**Figure 4:**
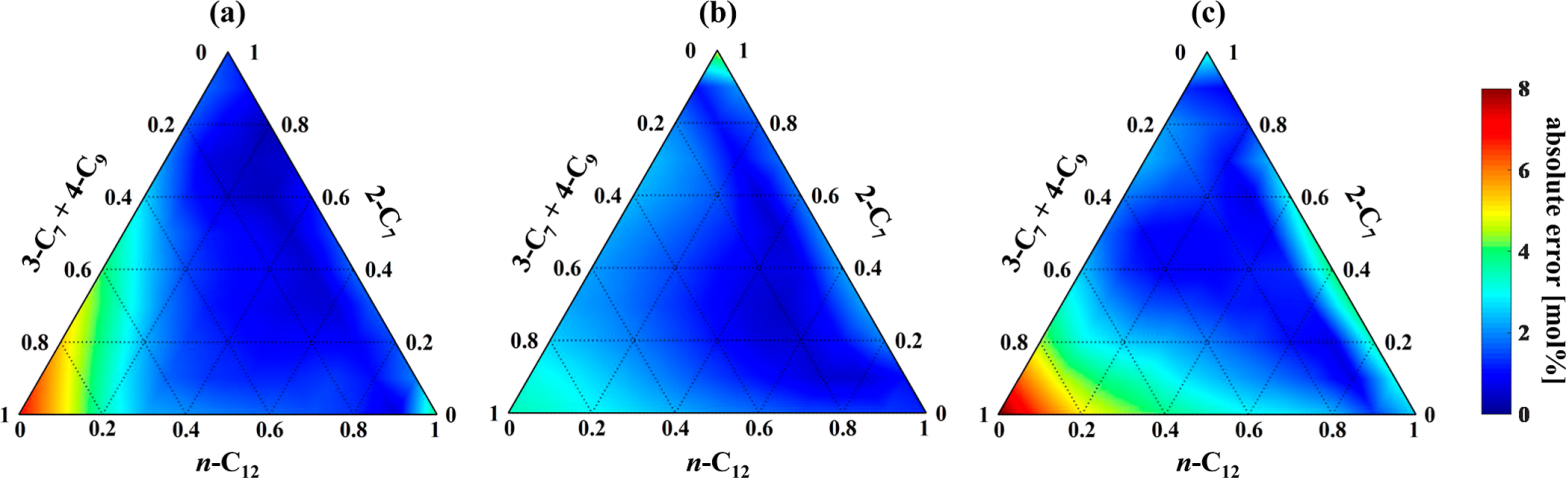
Distributions of the absolute errors for the PLSR estimation of the compositions of (a) *n*-C_12_, (b) 2-C_7_, and (c) 3-C_7_ + 4-C_9_ in the calibration mixtures.

As discussed previously, discrimination between 3-C_7_ and 4-C_9_ in porous titania was not possible, and hence, the mixtures of these four isomers in samples TM10–TM19 are considered as a ternary system, with 3-C_7_ and 4-C_9_ being treated as a single-component and the total composition of these two isomers being estimated in the PLSR analysis. The results of PLSR model calibration for this system are first presented. For each calibration mixture, the composition of the mixture estimated by the PLSR models has been compared with the known calibration composition, and the absolute error for each component of each calibration mixture is plotted in Figure 4. Figure 4a–c shows the components of *n*-C_12_, 2-C_7_, and 3-C_7_ + 4-C_9_, respectively. It is seen that for >90% of the composition space, the absolute errors are <5 mol % for all three components, suggesting accurate estimation of the PLSR models. However, larger errors are observed in estimating the *n*-C_12_ and 3-C_7_ + 4-C_9_ compositions at low *n*-C_12_ and high 3-C_7_ + 4-C_9_ compositions. This arises because *n*-C_12_ has only one cross-peak position at Δδ = ±0.40 ppm, which overlaps within the experimental error with the cross-peaks associated with the ^1^H attached to carbons [1, 2], [4, 5], and [9, 10] of 4-C_9_ and carbons [1, 2], [3, 4], and [7, 8] of 3-C_7_ (see Table S2). The RMSEC values for calibration mixtures were calculated as 2.4, 1.8, and 2.7 mol % for *n*-C_12_, 2-C_7_, and 3-C_7_ + 4-C_9_, respectively.

The results of PLSR estimation of the compositions of samples TM10–TM19 are listed in Table 1. It is seen that there is good agreement between the estimated and true compositions for all the bulk liquid and confined mixtures. The RMSEP values were calculated using the data presented in Table 1, and the values for *n*-C_12_, 2-C_7_, and 3-C_7_ + 4-C_9_ were obtained as 2.8, 1.3, and 3.0 mol %, respectively.

The analysis presented can be generalized to any mixtures of linear and monomethyl branched alkanes, with all monomethyl alkanes branched at carbon indices >3 treated as equivalent. For isomers with branching at carbon indices >3, it is assumed that the positions and intensities of cross-peaks do not change with the branching positions. Confirmation of the assumption was achieved using simulated spectra predicted by the open web-based software packages nmrshiftdb2^30^ and SPINUS.^31^

The PLSR analysis presented so far uses the calibration spectra based on the single-component spectra of monomethyl alkanes and *n*-alkanes and is therefore not applicable to multimethyl branched alkanes because the analysis assumes that one branched alkane molecule contains a single branching methyl group. However, it is now shown that the calibration spectra do allow the characterization of such mixtures in terms of the submolecular groups [CH_3_CH_2_, (CH_3_)_2_CH, and CH_2_CH(CH_3_)CH_2_] they contain. The PLSR models were calibrated to estimate the composition of submolecular groups, as detailed in the Supporting Information (section S2), where the calculation of the group compositions based on the molecular compositions is also presented. The absolute errors of the PLSR estimation of the group compositions of calibration mixtures are presented in Figure S3. The RMSEC values were calculated for each group in the calibration mixtures, yielding values of 0.9 mol % for all three groups. The results of the PLSR estimation of the group compositions of samples TM10–TM19 are listed in Table 1, based on which the RMSEPs were calculated as <1 mol % for all the three groups.

## Conclusions

In this work, 2D ^1^H DQF-COSY spectroscopy was applied to discriminate branched and linear alkanes. The main diagonal projection of the 2D data was used to characterize the compositions of mixtures of branched and linear alkanes confined within a porous titania catalyst support with the application of PLSR analysis. Two case studies are reported. First, mixtures of 2-methyl and linear alkanes were considered. Accurate PLSR estimation was achieved with an RMSEP of 1.4 mol % for 2-methyl alkane compositions. The 2-methyl alkane composition was also shown to be well predicted by a theoretical relationship which considered the relative intensity between the cross-peaks. In the second case, the method was applied to a more complicated system comprising *n*-C_12_, 2-C_7_, 3-C_7_, and 4-C_9_. The results showed that discrimination of 2-methyl and linear alkanes from other isomers in the mixtures was achieved. However, discrimination between monomethyl alkanes with the branching groups located at relatively central positions of alkyl chains was not achieved. PLSR models were applied to estimate the compositions of *n*-C_12_, 2-C_7_, and the total composition of 3-C_7_ and 4-C_9_ in the test mixtures, yielding RMSEP ≤3 mol % for all the components. The PLSR analysis was further extended to estimate the compositions of the submolecular groups CH_3_CH_2_, (CH_3_)_2_CH, and CH_2_CH(CH_3_)CH_2_ in the mixtures, resulting in RMSEP <1 mol % for all groups. The extended PLSR models calibrated for group compositions can be applied to determine the branching level in mixtures containing multimethyl branched alkanes. While the mixtures and their compositions considered in this work are of interest in FT synthesis, the method is considered generic and not limited to specific systems.

## Supporting Information Available

Details of the simulation of the spectra of calibration mixtures, of the implementation of PLSR calibration, and of error analysis; compositions of the test mixtures, and chemical shifts of coupled ^1^H nuclei that give rise to cross-peaks in the DQF-COSY spectra (PDF)
