# Supplementary material for: In Situ Characterization of Mixtures of Linear and Branched Hydrocarbons Confined within Porous Media Using 2D DQF-COSY NMR Spectroscopy
Source: Anal Chem. 2022 Feb 12;94(7):3135–41. doi: 10.1021/acs.analchem.1c04295 (PMC9098118; doi:10.1021/acs.analchem.1c04295)
Supplement: Supplementary file 1 — ac1c04295_si_001.pdf [file ac1c04295_si_001.pdf]

Supporting Information

In situ characterisation of mixtures of linear and branched hydrocarbons confined within porous media using 2D DQF-COSY NMR spectroscopy

Qingyuan Zheng†, Mick D. Mantle†, Andrew J. Sederman†,

Timothy A. Baart‡, Constant M. Guédon‡ and Lynn F. Gladden*†

†Department of Chemical Engineering and Biotechnology, University of Cambridge, West Cambridge Site, Philippa Fawcett Drive, Cambridge CB3 0AS, UK

‡ Shell Global Solutions International B.V., Grasweg 31, Amsterdam 1031 HW, The Netherlands

Table of contents

Section S1. Simulation of 1D anti-diagonal spectra of calibration mixtures S2
Section S2. Calibration of PLSR models S3
Section S3. Error analysis S5
References S6
Figure S1. Validation of simulated 1D anti-diagonal spectra S7
Figure S2. Simulated 1D anti-diagonal spectra of calibration mixtures S8
Figure S3. Absolute errors for PLSR estimation of the group compositions S9
Table S1. Gravimetrically-determined mole fractions of test mixtures S10
Table S2. Chemical shifts of coupled ^{1}H nuclei S11

S1. Simulation of 1D anti-diagonal spectra of calibration mixtures

The calibration of PLSR models requires the spectral data **X** associated with mixtures of known compositions **Y**. In this work, the 1D anti-diagonal spectra of calibration mixtures were simulated by linear combination of the spectra of the individual species contained in the mixtures, according to the mixture composition. The simulation of mixture spectra is described as follows.

Given that the NMR signal of a species is proportional to the number of ^{1}H nuclear spins associated with that chemical species present in the mixture, the NMR data in the time domain, namely the free induction decay (FID), of a mixture is calculated as:

$$\mathbf{FID}_{\mathrm{mix}} = \sum_i x_i\, \mathbf{FID}_i, \qquad \text{(S1)}$$

where $\mathbf{FID}_{\mathrm{mix}}$ is the simulated FID of the mixture of a reference number of moles, x_i is the mole fraction of species i and $\mathbf{FID}_i$ is the FID of single-component species i of the reference number of moles. The experimentally measured FIDs of the single-component species will have different numbers of moles in the samples due to differing molar densities and sample volumes of the single-component samples. Therefore the experimentally measured FIDs were scaled to the reference number of moles using the known molar densities of the single components and the volume of the samples, where the volume was calculated from a 1D MRI profile of the sample. After obtaining $\mathbf{FID}_{\mathrm{mix}}$, the time domain data were processed to yield the 1D anti-diagonal spectra as described in the main text.

The 1D anti-diagonal spectra of bulk liquid samples TM10-TM15 (Table S1) were simulated based on the mixture compositions and are compared with the spectra experimentally measured from these samples in Fig. S1. It is observed that in general the simulated spectra are in good agreement with the measured spectra with the largest error observed at $\Delta\delta$ = 0 ppm corresponding to the peak from the main diagonal in the 2D spectra. The relatively large error for this peak could be due to the fact that the suppression of signal for peaks on the main diagonal in the DQF-COSY experiment is sensitive to local magnetic field inhomogeneity which is slightly different for different samples. For cross peaks located at $\Delta\delta \neq 0$ ppm, the relative errors between the cross peak intensities of the simulated and measured spectra were calculated for the samples in Fig. S1 and an average value of these errors was 6%.

S2. Calibration of PLSR models

The PLSR method is introduced briefly as follows. In PLSR, the regression relationship between spectral matrix **X** ($n \times m$, where n is the number of calibration samples and m is the number of data points in the anti-diagonal spectra) and composition matrix **Y** ($n \times p$, where p is the number of mixture components or sub-molecular groups) is identified by finding the principal components (PCs) of **X** and **Y** with the covariance between these PCs maximised[1,2]. The model structure of PLSR can be written as:

$$\mathbf{X} = \mathbf{TP} + \mathbf{E}, \tag{S2}$$

$$\mathbf{Y} = \mathbf{TQ} + \mathbf{F}, \tag{S3}$$

where **T** ($n \times b$, b is the number of PCs for **X**) is the score matrix of **X**, **P** ($b \times m$) and **Q** ($b \times p$) are the loading matrices of **X** and **Y** respectively, and **E** and **F** are the residual matrices. The score and loading matrices are obtained in model calibration. Estimation of the composition $\hat{\mathbf{Y}}$ with the spectra $\mathbf{X}_{test}$ of test samples is achieved as follows:

$$\hat{\mathbf{Y}} = \mathbf{X}_{test}\hat{\mathbf{b}}, \tag{S4}$$

where $\hat{\mathbf{b}}$ is the matrix of regression parameters, obtained using the score and loading matrices. The PLSR models were calibrated using the calibration spectral data generated from the data of single-component bulk liquid of n-C_{12}, 2-C_7, 3-C_7 and 4-C_9 based on known mixture compositions following the method described in section S1. The spectral data and compositions of calibration mixtures constitute the matrices **X** and **Y**, and the creation of these two matrices are now described.

The composition matrix **Y** for a binary system was created with the composition of one component increasing from 0 to 1 in 10 steps, yielding a 11×2 matrix $\mathbf{Y}_2 = [\mathbf{y}, 1\text{-}\mathbf{y}]$ where **y** is the 11×1 vector [0;0.1;0.2;…0.9;1]. The $\mathbf{Y}_2$ matrix was used in generating the calibration spectral data for the mixtures of 2-methyl and linear alkanes using the spectral data of the bulk liquids of pure n-C_{12} and 2-C_7, yielding 11 calibration spectra. For the mixtures of n-C_{12}, 2-C_7, 3-C_7 and 4-C_9, the composition matrix $\mathbf{Y}_4$ for the 4-component system is required. To obtain $\mathbf{Y}_4$, the composition matrix for a ternary system $\mathbf{Y}_3$ was first created with the first component of the ternary system varying according to **y**. Denoting

the elements of **y** as y_i, for each value of y_i = 0-0.9 for the first component, the compositions of the second and third components were described by the 11×2 matrix $(1-y_i)\mathbf{Y}_2$. This resulted in 110 different compositions for the ternary system. With one more composition corresponding to y_i = 1 added to the composition matrix, the 111×3 matrix $\mathbf{Y}_3$ was obtained. To create the composition matrix $\mathbf{Y}_4$ for a quaternary system, similar to the creation of $\mathbf{Y}_3$, the composition of the first component of the quaternary system was described by **y**. For each value of y_i = 0-0.9, the compositions of the other 3 components were described by the 111×3 matrix $(1-y_i)\mathbf{Y}_3$, resulting in 1110 different compositions for the quaternary system. Together with one additional composition with y_i = 1, the 1111×4 matrix $\mathbf{Y}_4$ was obtained. As discussed in the main text the 4-component mixtures were treated as a ternary system of *n*-C_{12}, 2-C_7 and a joint component 3-C_7+4-C_9. Therefore, to obtain the composition matrix **Y** that was used for PLSR calibration, the columns of **Y** corresponding to *n*-C_{12} and 2-C_7 were obtained directly from those of $\mathbf{Y}_4$ for the same species. The column of **Y** corresponding to the component 3-C_7+4-C_9 was calculated by adding the $\mathbf{Y}_4$ columns corresponding to these 2 species.

To obtain the spectral matrix **X** for PLSR calibration, a 1D anti-diagonal spectrum was simulated based on each of the 1111 compositions described by $\mathbf{Y}_4$ and the simulated spectra are shown in Fig. S2. For mixtures of *n*-C_{12} and 2-C_7, the spectral data at $\Delta\delta$ = 0.40-0.65 ppm were used as the spectral matrix **X**. For the 4-component systems, the spectral data at $\Delta\delta$ = 0.2-0.8 ppm were used as **X**.

The simulated spectra **X** and the corresponding compositions **Y** were then used to calibrate PLSR models. The calibration was implemented using the NIPALS algorithm[3]. The optimal numbers of principal components of the PLSR models were determined using the method of Gowen et al.[2]. For mixtures of *n*-C_{12} and 2-C_7, two principal components were used for both species. For mixtures of *n*-C_{12}, 2-C_7, 3-C_7 and 4-C_9, the optimal numbers of principal components were determined as 3, 4 and 4 for estimating the composition of 2-C_7, the total composition of 3-C_7 and 4-C_9 and the composition of *n*-C_{12}, respectively.

To calibrate PLSR models for estimating the compositions of groups CH_3CH_2, $(CH_3)_2CH$ and $CH_2CH(CH_3)CH_2$, the same spectral matrices **X** described earlier were used along with a group composition matrix $\mathbf{Y}_g$ obtained from $\mathbf{Y}_2$ or $\mathbf{Y}_4$ as follows. The calculation of group compositions is based on molecular structures such that a *n*-C_{12} molecule contributes two CH_3CH_2 groups, a 2-C_7 molecule contributes one $(CH_3)_2CH$ group and one CH_3CH_2 group,

and a 4-C_9 molecule contributes one $CH_2CH(CH_3)CH_2$ group and two CH_3CH_2 groups. A 3-C_7 molecule contributes one $CH_2CH(CH_3)CH_2$ group and one CH_3CH_2 group; this is because the second CH_3CH_2 with carbon indices [1, 2'] does not contribute to cross peak intensity at $\Delta\delta$ = ± 0.40 ppm (Table S2). In this work, only the CH_3CH_2 group associated with cross peaks at $\Delta\delta$ = ± 0.40 ppm is considered. Denoting the composition vectors of n-C_{12}, 2-C_7, 3-C_7 and 4-C_9 as $\mathbf{y}_{n-\mathrm{C}_{12}}$, $\mathbf{y}_{2-\mathrm{C}_7}$, $\mathbf{y}_{3-\mathrm{C}_7}$ and $\mathbf{y}_{4-\mathrm{C}_9}$ respectively, the column of $\mathbf{Y}_\mathrm{g}$ corresponding to the CH_3CH_2 group that leads to the cross peaks at $\Delta\delta$ = ± 0.40 ppm was calculated as $2\mathbf{y}_{n-\mathrm{C}_{12}}$+$\mathbf{y}_{2-\mathrm{C}_7}$+$\mathbf{y}_{3-\mathrm{C}_7}$+$2\mathbf{y}_{4-\mathrm{C}_9}$ where the multiplication constants of composition vectors indicate the number of CH_3CH_2 groups in a given molecule. The columns of $\mathbf{Y}_\mathrm{g}$ corresponding to $(CH_3)_2CH$ and $CH_2CH(CH_3)CH_2$ groups were obtained as $\mathbf{y}_{2-\mathrm{C}_7}$ and $\mathbf{y}_{3-\mathrm{C}_7}$+$\mathbf{y}_{4-\mathrm{C}_9}$, respectively.

S3. Error analysis

The error of the PLSR estimation was evaluated using *RMSE* defined by:

$$RMSE = \sqrt{\frac{1}{N}\sum_{i=1}^{N}(\hat{x}_i - x_i)^2}, \tag{S5}$$

where N is the number of samples, $\hat{x}_i$ and x_i are the estimated and actual mole fractions respectively for sample i. When calculated for one sample N = 1, *RMSE* becomes the absolute error = $|\hat{x}_i - x_i|$.

The standard error of the PLSR estimated compositions for each sample reported in Table 1 was calculated from 2–3 measurements of the same sample.

References

(1) Wold, S.; Sjöström, M.; Eriksson, L. PLS-Regression: A Basic Tool of Chemometrics. *Chemom. Intell. Lab. Syst.* **2001**, *58*, 109–130.

(2) Gowen, A. A.; Downey, G.; Esquerre, C.; O’Donnell, C. P. Preventing Over-Fitting in PLS Calibration Models of Near-Infrared (NIR) Spectroscopy Data Using Regression Coefficients. *J. Chemom.* **2011**, *25*, 375–381.

(3) Geladi, P.; Kowalski, B. R. Partial Least-Squares Regression: A Tutorial. *Anal. Chim. Acta* **1986**, *185*, 1–17.

(4) Spectral Database for Organic Compounds SDBS https://sdbs.db.aist.go.jp.

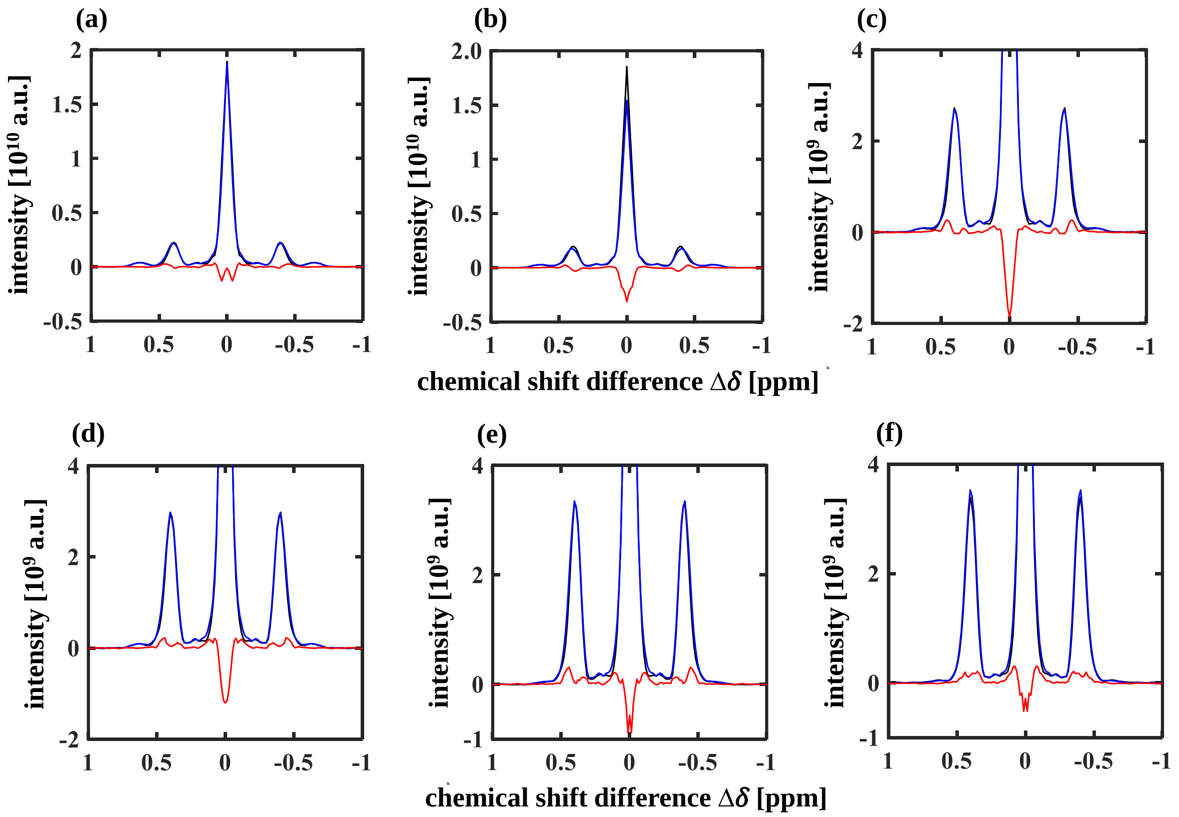

Figure S1. Comparison between the simulated and measured 1D anti-diagonal spectra. The results for samples TM10-TM15 in Table S1 are presented in (a)-(f), respectively. The measured and simulated spectra are shown as black and blue, respectively. The difference between the simulated and measured spectra is shown in red.

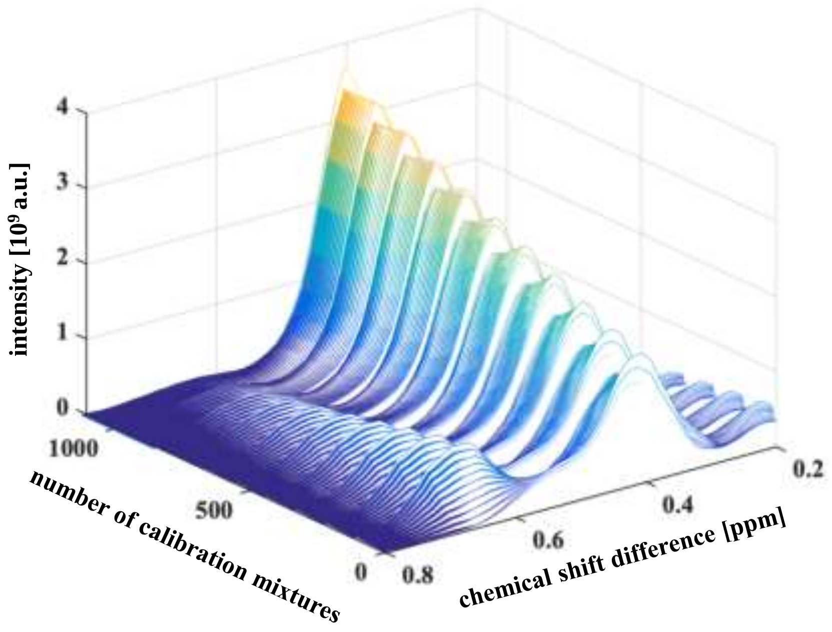

Figure S2. Simulated 1D anti-diagonal spectra of calibration mixtures consisting of *n*-C_{12}, 2-C_7, 3-C_7 and 4-C_9. Only the cross peaks used in the PLSR analysis are shown ($\Delta\delta$ = 0.2-0.8 ppm).

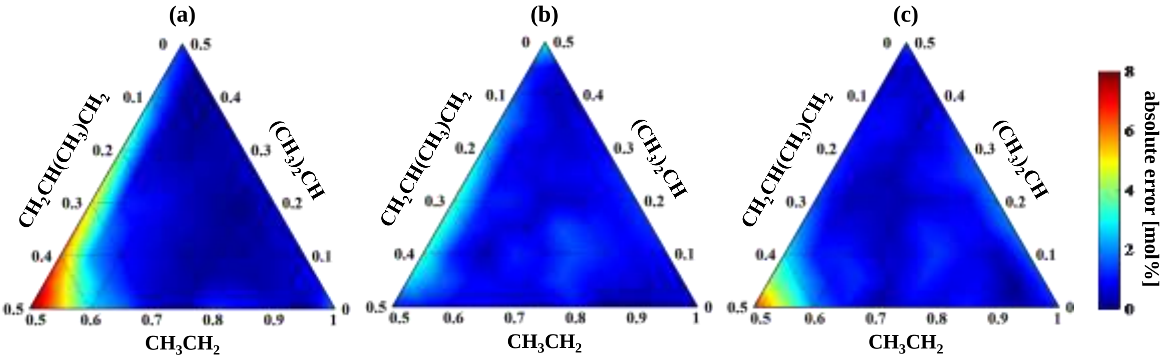

Figure S3. Distributions of the absolute errors for PLSR estimation of the compositions of groups (a) CH_3CH_2, (b) $(CH_3)_2CH$, and (c) $CH_2CH(CH_3)CH_2$ in calibration mixtures.

Table S1. Gravimetrically-determined mole fractions of test mixtures (TMi) to validate the PLSR models. Whether the liquid mixture is confined within the porous titania or in the bulk liquid state is also indicated.

	**mole fractions, *x* [mol%]**							
**samples**	**2-C_7**	**2-C_9**	**3-C_7**	**4-C_9**	***n*-C_{10}**	***n*-C_{12}**	***n*-C_{16}**	**state**
TM1	5.6	0	0	0	0	94.4	0	confined
TM2	9.8	0	0	0	0	90.2	0	confined
TM3	15.4	0	0	0	0	84.6	0	confined
TM4	20.0	0	0	0	0	80.0	0	confined
TM5	40.0	0	0	0	0	60.0	0	confined
TM6	60.0	0	0	0	0	40.0	0	confined
TM7	100.0	0	0	0	0	0	0	confined
TM8	10.8	0	0	0	0	44.5	44.7	confined
TM9	0	10.4	0	0	89.6	0	0	confined
TM10	33.4	0	33.7	0	0	32.9	0	bulk
TM11	25.2	0	25.3	24.9	0	24.5	0	bulk
TM12	8.6	0	19.3	12.2	0	60.0	0	bulk
TM13	9.4	0	11.8	8.6	0	70.1	0	bulk
TM14	3.8	0	10.7	7.5	0	78.0	0	bulk
TM15	3.7	0	4.6	3.8	0	87.8	0	bulk
TM16	7.9	0	19.8	12.4	0	59.9	0	confined
TM17	8.9	0	13.3	8.2	0	69.6	0	confined
TM18	4.1	0	9.8	6.6	0	79.4	0	confined
TM19	2.8	0	4.5	2.7	0	90.0	0	confined

Table S2. Chemical shifts of ^{1}H nuclei involved in the *J*-coupling that results in the cross peaks (XP) shown in Fig. 1. The first two columns present the molecules and their schematics. The third column lists the coupling ^{1}H where subscripts i and j denote the indices of carbons to which the ^{1}H are attached. The fourth column lists the chemical shifts of coupling ^{1}H. The fifth column presents the chemical shift difference of coupling ^{1}H which indicates the positions of cross peaks in the anti-diagonal spectra.

molecules		$[i, j]$	$[\delta_i, \delta_j]$ [ppm]	$\Delta\delta_{i-j}^{XP}$ [ppm]
n-C_{12}	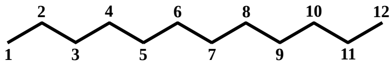	[1, 2]	[0.87, 1.27]	± 0.40
		[11, 12]	[1.27, 0.87]	± 0.40
2-C_7	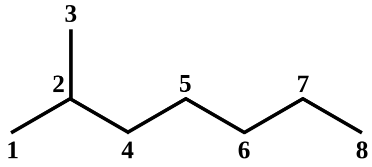	[1, 2]	[0.87, 1.52]	± 0.65
		[2, 3]	[1.52, 0.87]	± 0.65
		[2, 4][a]	[1.52, 1.18]	± 0.34
		[4, 5]	[1.18, 1.28]	± 0.10
		[7, 8]	[1.28, 0.88]	± 0.40
3-C_7	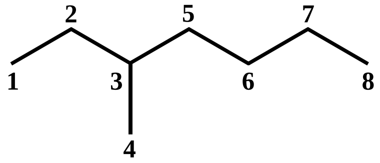	[1, 2]	[0.86, 1.33]	± 0.47
		[1, 2′][b]	[0.86, 1.08]	± 0.22
		[2, 2′][b]	[1.33, 1.08]	± 0.25
		[2, 3]	[1.33, 1.37]	± 0.04
		[2′, 3]	[1.08, 1.37]	± 0.29
		[3, 4]	[1.37, 0.82]	± 0.55
		[3, 5]	[1.37, 1.23[c]]	± 0.14
		[3, 5′][b]	[1.37, 1.13]	± 0.24
		[5, 5′][b]	[1.23[c], 1.13]	± 0.10
		[5, 6]	[1.23[c], 1.26]	± 0.03
		[5′, 6][b]	[1.13, 1.26]	± 0.13
		[7, 8]	[1.26, 0.87]	± 0.39
4-C_9	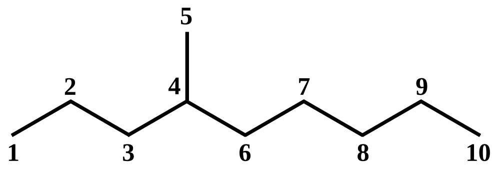	[1, 2]	[0.90, 1.30]	± 0.40
		[2, 3]	[1.30, 1.22[c]]	± 0.08
		[2, 3′][b]	[1.30, 1.07[c]]	± 0.23
		[3, 3′][b]	[1.22[c], 1.07[c]]	± 0.15
		[3, 4]	[1.22[c], 1.42]	± 0.20
		[3′, 4][b]	[1.07[c], 1.42]	± 0.35
		[4, 5]	[1.42, 0.87]	± 0.55
		[4, 6]	[1.42, 1.24[c]]	± 0.18
		[4, 6′][b]	[1.42, 1.10[c]]	± 0.32
		[6, 6′][b]	[1.24[c], 1.10[c]]	± 0.14
		[6, 7]	[1.24[c], 1.30]	± 0.06
		[6′, 7][b]	[1.10[c], 1.30]	± 0.20
		[9, 10]	[1.30, 0.90]	± 0.40

[a]not visible in Fig. 1b due to low signal intensity
[b]the prime symbol indicates the ^{1}H attached to the same carbon but with different chemical shifts
[c]chemical shift obtained from the SDBS database[4]
